# An architecture for genomics analysis in a clinical setting using Galaxy and Docker

**DOI:** 10.1093/gigascience/gix099

**Published:** 2017-10-18

**Authors:** W Digan, H Countouris, M Barritault, D Baudoin, P Laurent-Puig, H Blons, A Burgun, B Rance

**Affiliations:** 1 University Hospital Georges Pompidou, HEGP, Department of Medical Informatics, AP-HP, INSERM, Centre de Recherche des Cordeliers, UMRS 1138, Université Sorbonne Paris Cité, University Paris-Descartes, Paris, France; 2 University Hospital Georges Pompidou, HEGP, Department of Biochemistry, Pharmacogenetics and Molecular Oncology, AP-HP, Paris, France, University Paris-Descartes; 3 INSERM UMR-S1147, CNRS SNC 5014, Université Sorbonne Paris Cité, Paris, France

**Keywords:** Galaxy, ReGaTE, Docker, reproducibility

## Abstract

Next-generation sequencing is used on a daily basis to perform molecular analysis to determine subtypes of disease (e.g., in cancer) and to assist in the selection of the optimal treatment. Clinical bioinformatics handles the manipulation of the data generated by the sequencer, from the generation to the analysis and interpretation. Reproducibility and traceability are crucial issues in a clinical setting. We have designed an approach based on Docker container technology and Galaxy, the popular bioinformatics analysis support open-source software. Our solution simplifies the deployment of a small-size analytical platform and simplifies the process for the clinician. From the technical point of view, the tools embedded in the platform are isolated and versioned through Docker images. Along the Galaxy platform, we also introduce the AnalysisManager, a solution that allows single-click analysis for biologists and leverages standardized bioinformatics application programming interfaces. We added a Shiny/R interactive environment to ease the visualization of the outputs. The platform relies on containers and ensures the data traceability by recording analytical actions and by associating inputs and outputs of the tools to EDAM ontology through ReGaTe. The source code is freely available on Github at https://github.com/CARPEM/GalaxyDocker.

## Introduction

The rise of genomics in recent years has impacted virtually every domain of medicine. The reductions of the cost of sequencing and the popularization of the technologies have contributed to the large adoption of next-generation sequencing (NGS) both in research and clinical settings. NGS results have become essential to medical decision-making, similar to pathology results or any other clinical information collected during patient care. In oncology, sequencing technologies have had a large influence on therapeutic choices in almost all types of cancers, including carcinomas (lung, colon, skin), sarcomas, and brain (gliomas) or blood (leukemias, lymphomas, myelomas) cancers. Clinicians use NGS to identify actionable mutations (such as epidermal growth factor receptor (EGFR) for pulmonary carcinoma, or BRAF in the case of skin cancer) and adapt the treatment for optimal efficacy [1–7].

With the increasing role of NGS, the importance of bioinformatics has also risen. Bioinformaticians handle the manipulation of the massive amount of produced data, the development of dedicated analysis pipelines, the traceability of analysis, and so forth. Most large research structures have bioinformatics research groups. However, the massive introduction of NGS into routine care, like the perspective of at least 5 targeted (or exome) sequencing methods for the diagnosis and follow-up of cancer cases [8], requires automated pipelines for acquiring, storing, organizing, and analyzing biological data that support the delivery of patient care. In such a setting, we differentiate *clinical bioinformatics* that mainly handle the problems related to data, as well as their production, manipulation, and storage, and *research bioinformatics* dedicated to the development of new analytical methods. Indeed, bioinformatics in a clinical environment is substantially different from *research* bioinformatics: the variety of analyses is limited but largely repeated, and time and the resources to provide a result are constrained (in personal as well as material terms).

In many hospitals, the analysis of NGS results still relies on tedious manual expertise to use a series of commercial and open-source tools both online and offline. The molecular biologist performs the quality control and analysis of NGS results, together with the clinician. Often, the analysis and production of the curated results are the bottleneck of the overall process. In addition, one major concern in bioinformatics, especially when the NGS results are used to make clinical decisions, is traceability and reproducibility. Sandve et al. [9] have identified good practices that would contribute to ensuring research quality. Among these rules, several apply to clinical bioinformatics: keeping track of how the results were produced, avoiding manual data manipulation, archiving the versions of all the programs, storing raw data and intermediate results, linking textual statements to raw results. We have developed our system with these recommendations in mind.

A new technology has appeared in recent years that can be of great help in a clinical bioinformatics setting. Docker [10] is a Linux-based open-source technology, used on a daily basis by thousands if not millions of users in the world. Docker is a container technology that isolates tools from the operating system of the host server. Among other features, it allows users to deploy tools in a quick and reproducible manner. Different containers can run different versions of the same tools or library without any mutual interference.

We designed an approach based on Docker and Galaxy [11], the popular bioinformatics analysis support open-source software, that (1) is easy to manage and transparent to the user, (2) enables traceability of the result, (3) enables reproducible analysis, (4) can be deployed in a clinical setting with limited bioinformatics expertise and resources, (5) reduces the burden of manual analysis and data manipulation for molecular biologists, and (6) shortens the delay between the sample processing and the production of a clinically relevant result.

### Related works

#### Workflow management systems

The development of high-throughput methods in molecular biology has considerably increased the volume of molecular data produced daily by biologists. Many analytical scripts and software have been developed to assist biologists and clinicians in their tasks. Commercial and open-source solutions have emerged, allowing the user to combine analytical tools and build pipelines using graphical interfaces. In addition, workflow management systems (such as Taverna [12], Galaxy [11], SnakeMake [13], NextFlow [14]) also ensure the traceability and reproducibility of the analytical process. The efficient use of a workflow management system remains limited to trained bioinformaticians.

#### Docker and Galaxy

Docker provides a standard way to supply ready-to-use applications, and it's beginning to be a common way to share works [15–22]. In Aranguren and Wilkinson [23], the authors make the assumption that the reproducibility could be implemented at 2 levels: (1) at the Docker container level: the encapsulation of a tool with all its dependencies would ensure the sustainability, traceability, and reproducibility of the tool; and (2) at the workflow level: the reproducibility is ensured by Galaxy workflow definition. They developed a containerized Galaxy Docker platform in the context of the OpenLifeData2SADI research project. In Kuenzi et al. [16], the authors distribute a Galaxy Docker container hosting a tool suite called APOSTL, which is dedicated to proteomics analysis of mass spectrometry data. They implemented R/Shiny [24] environments inside Galaxy.

Grüning et al. [17] provide a standard Dockerized Galaxy application that can be extended in many flavors [18, 25, 26]. Some Galaxy Dockerized applications already exist in containers, e.g., deepTools2 [18].

The integration of new tools in Galaxy can be simplified by applications generating configuration files that are near-ready for integration [27, 28]. We propose a similar tool (the DockerTools2Galaxy script), dedicated to Dockerized tools.

In this article, we present our architecture to deploy a bioinformatics platform in a clinical setting, leveraging the worldwide-known bioinformatics workflow management solution Galaxy and Docker virtualization technology, standardized bioinformatics application programming interfaces (APIs), and graphical interfaces developed in R SHINY.

## Methods

### Galaxy

**Figure 1:**
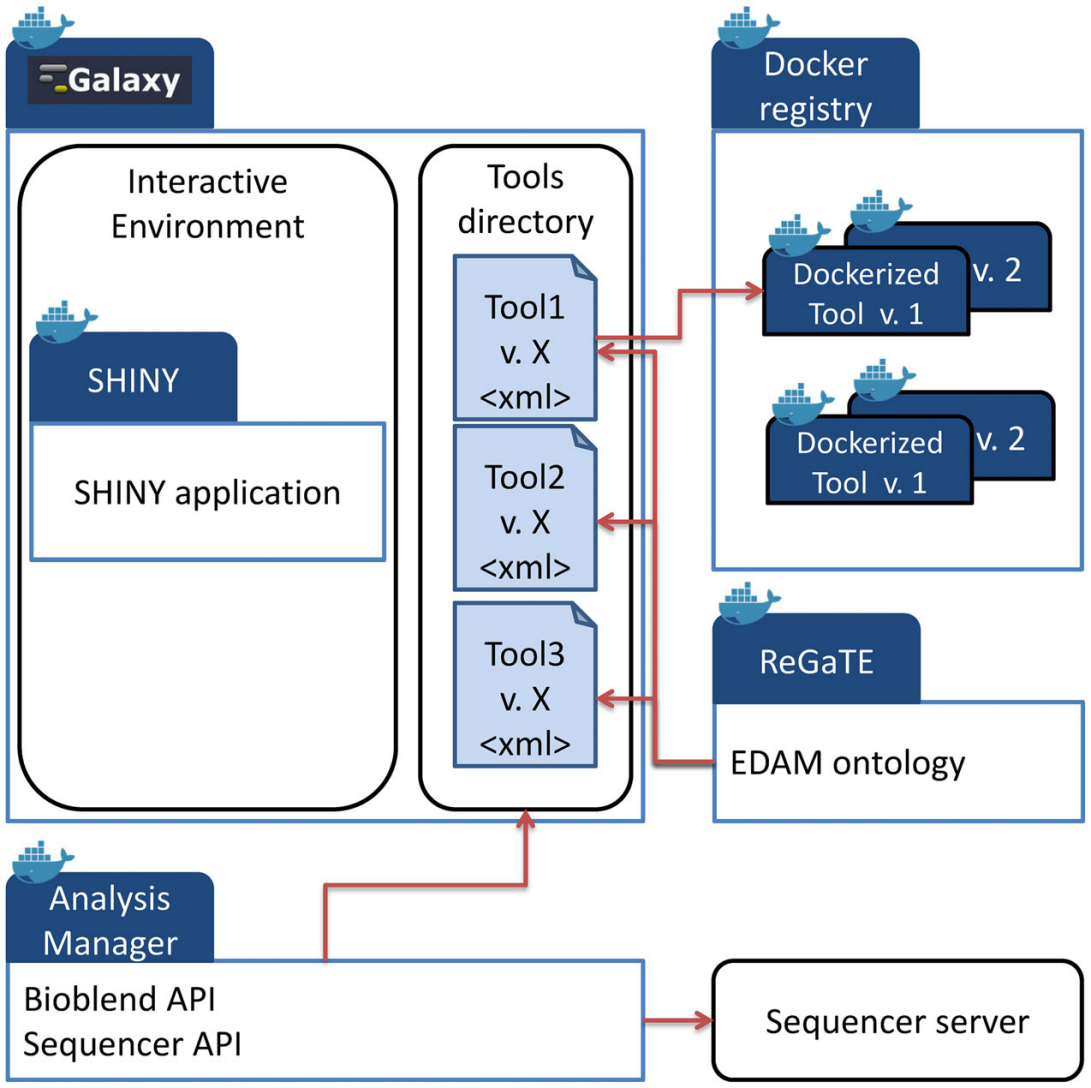
Overall architecture of our Galaxy Docker solution. Each tool comes as an independent Docker image.

We leveraged the Galaxy version (Galaxy, RRID:SCR_006281) distributed as a Docker image by B. Grüning. We integrated a local Docker registry to store and organize all our Galaxy tools (see Fig. 1). The registry also handles the versioning of the tools. We added to Galaxy 2 volumes to manipulate input and output data and to keep the reference genomes and other associated data. Furthermore, we configured the Galaxy instance to handle authentication using the Lightweight Directory Access Protocol (LDAP) for better integration in our information system.

### Dockerized tools and registry

In our proposed architecture, the tools themselves are Dockerized. We developed homemade solutions and adapted standard NGS tools. We propose a Python script to help create Docker images of tools for Galaxy. The script takes a list of Docker images as input and generates for each tool a Galaxy-ready configuration (xml) file. A complete runnable example is provided on the github repository of the project.

### SHINY application

**Figure 2:**
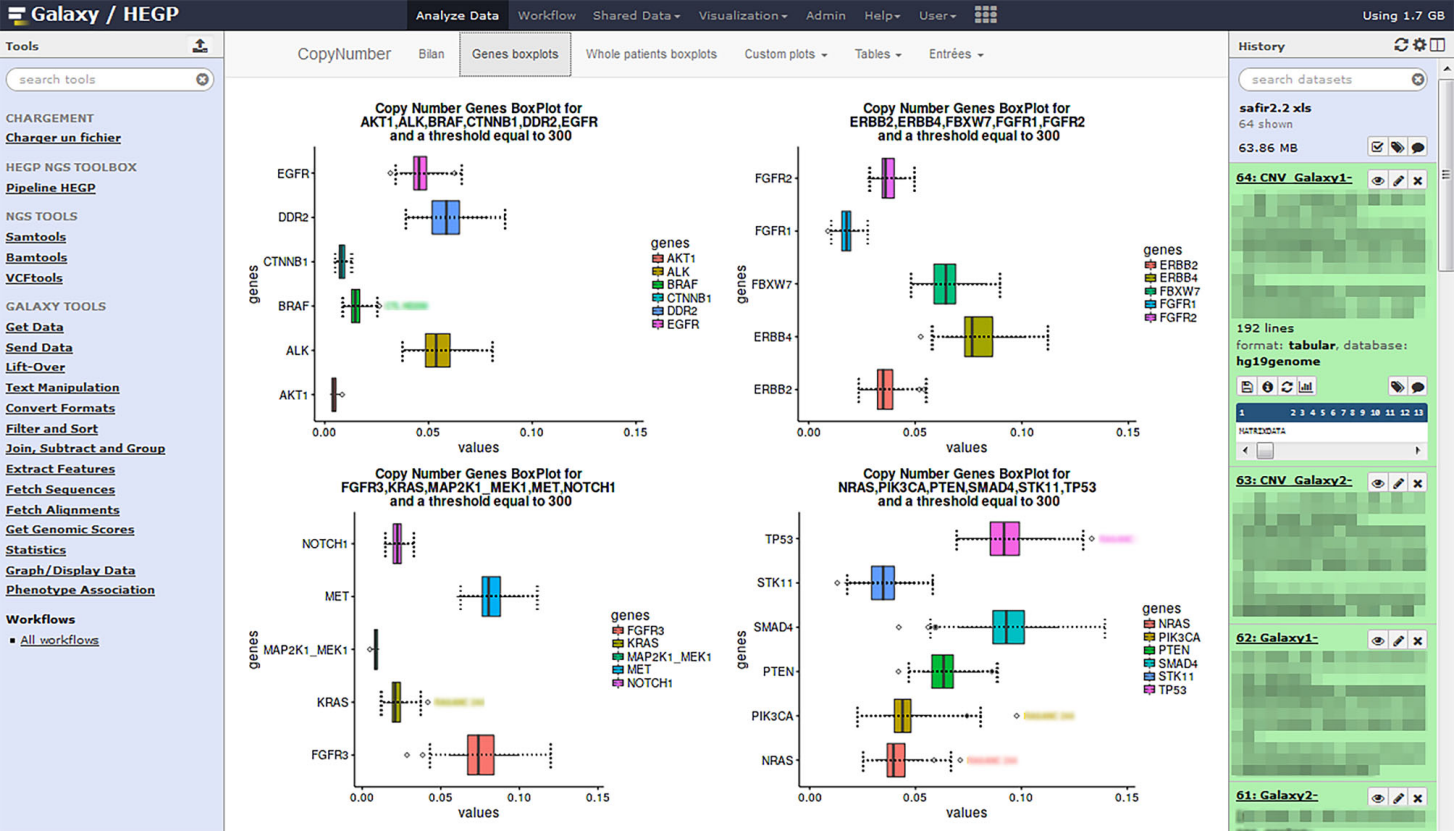
The R/Shiny interface embedded inside Galaxy. In this example, the Shiny interface provides boxplot visualizations of gene coverage (outliers represent patients presenting amplification or deletion for these genes).

We developed a Dockerized SHINY application (Shiny, RRID:SCR_001626) to simplify the exploration of users’ results. Our SHINY interface is included in Galaxy as a new interactive environment (see Fig. 2). The application is based on RStudio (RStudio, RRID:SCR_000432) [29] and iobam [30]. We took advantage of R packages including ggplot2 (ggplot2, RRID:SCR_014601) [31] for graphics and DT tables [32] for advanced table representation.

### Analysis manager

**Figure 3:**
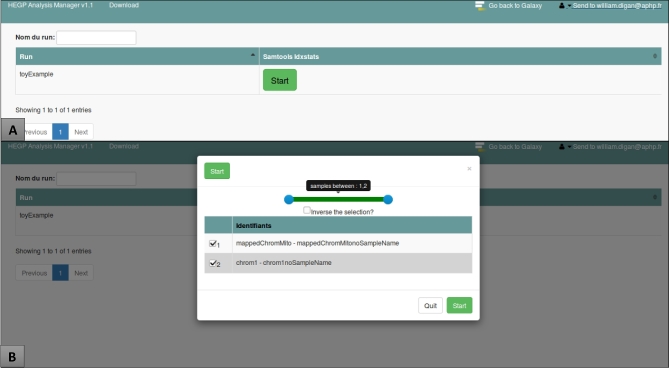
The AM “one click” interface: after registering to Galaxy, the user is able to use the AM. **(A)** This window lists the datasets ready for analysis. By clicking on the “Start” button, a pop-up window will appear. **(B)** The user can select specific files (here BAM files) and start an analytic workflow within Galaxy (in this example, the Samtools idstat analysis) for each of the files.

The AnalysisManager (AM) aims at simplifying the task of the biologists and reducing the burden of routine analysis. The AM enables the launch of common analytical processes in a single click (see Fig. 3). Like most of the components of our architecture, the AM is based on Docker. We developed a Django web application (in Python) using Bootstrap [33] framework.

The AM handles 3 aspects of data treatment: (1) retrieving and backing-up the raw data from the sequencer using user-defined sample names (in addition to the sequencer barcodes); we leveraged the sequencer APIs to ensure compatibility across update releases; (2) running a user-selected Galaxy workflow, and adding the workflow to a Galaxy user's history; we used the BioBlend API (BioBlend Library, RRID:SCR_014557) [34] to connect our application to Galaxy; (3) finally, the AM backs up processed output files and sends an automated email to alert the user that their analysis is ready. The AM uses a PostgresSQL database to store the user operations. Asynchronous Tasks jobs are performed with Celery and Redis [35, 36].

ReGaTE [37] aims at automating the publication of Galaxy tools in the ELIXIR bio-tools registry by generating EDAM ontologies annotations [29]. The EDAM ontology goal is to describe and characterize bioinformatics operations such as data types and format. We choose to Dockerize ReGaTE and to include it in our infrastructure. We leverage ReGaTE to produce metadata associated with every tool from the Galaxy instance, used by the reproducibility logs.

## Results

### General architecture

We extended the Galaxy application distributed as a Docker container by Grüning [17]. This version follows closely the development cycle of the official Galaxy release. We adapted this image by adding our own tools, distributed as Docker images within Galaxy (i.e., Docker in Docker). For the user, the underlying architecture is transparent. The biologist would select a tool as they usually do and start an analysis. We also added graphical interfaces to some of our tools by creating graphical interfaces in R SHINY as Galaxy-interactive environments. The detailed architecture of our proposed solution is presented in the “Methods” section.

### Distribution

The Dockerized Galaxy embedding examples of Dockerized tools (Docker in Docker), the ReGaTE [37] traceability metadata creation system, configuration files, and code, and the AnalysisManager module (see Fig. 3) connecting a sequencer to the services of the Galaxy platform directly can be found online [38].

The tools themselves are released by their original developers.

### Graphical interface in R SHINY

We leverage the R SHINY [24] applications to create graphical interfaces for some of our tools. Shiny R applications take advantage of the profusion of visualization libraries. SHINY applications require no coding capabilities from their users and allow the sharing of advanced visualization or statistical analysis with a layman user. The interface is embedded in Galaxy as a new Interactive Environment (see Fig. 2).

### A recipe to integrate additional tools

We implemented a tool to ease the integration of Dockerized tools in Galaxy. DockerTools2Galaxy generates configuration files ready for integration in Galaxy, given a Dockerized tool as an input. Using variables predefined in the Dockerized tool, DockerTools2Galaxy produces an xml configuration file.

## Discussion

We have developed a system ensuring that the recommendations regarding quality insurance for NGS pipelines are followed. The system is already in use at European Hospital Georges Pompidou (HEGP) for cancer patients and has reduced the workload both for bioinformaticians and for molecular biologists.

### Comparison with other works

Cluster Flow [39] is a workflow manager using command lines. The tool requires programming skills and is not appropriate to the typical clinician end user. Knime4NGS [40] is a graphical interface, but it is not yet available as a Docker image for rapid testing or deployment. NEAT [41] seems to have a smaller developer community than Galaxy. The last update made to their public release was 2 years ago in 2015. JMS [42], a workflow management platform, focuses mostly on high-performance computing. Our architecture aims mainly at small structures.

Our work mostly compares to the different flavors of Galaxy implementations. We leveraged the Docker Galaxy image from Grüning et al. [17]. In a recent work [43], Grüning et al. attempted to ensure the deployment of a hybrid platform that allows raw data exploration through an interactive platform (Jupyter). They also define 2 main stages of NGS analysis. The first involves processing routinely some raw data with a set of defined/known tools and parameters that can be automatized and standardized. The second stage involves tools that need to be self-customized. This stage is more self-exploratory. Our work belongs to the first stage. We made it possible to plug our Galaxy instance into a network that already included an NG-sequencer and that manages the main steps of standard and reproducible data processing until the final analysis, making the results available inside Galaxy.

### Galaxy toolshed and Dockerized tools

The Galaxy toolshed provides an efficient way to share tools. However, it may be difficult to maintain and be able to run different versions of a same tool, especially if the various versions make use of different versions of libraries. Docker provides a great solution to this problem. In addition, the containerization can reduce drastically the burden of the installation of complex tools. Our proposition could be a first step toward accepting Dockerized tools within the Galaxy toolshed.

### Limitations

#### Generalization

So far, the AM has been connected to a single type of sequencer. The addition of a connector to other sequencers can be done by replacing the Ion-torrent API calls by the API calls from the local sequencer.

#### Integration to the information system

We were not yet able to integrate the platform with the laboratory management information system. While no connectors are implemented, we formatted the output to simplify the export process (through copy/paste).

#### Data privacy

The platform is deployed as a clinical tool within the hospital. The platform is not accessible from outside the hospital and requires strictly controlled accounts. However, the system was not designed to respect HIPAA or European privacy regulation by itself.

#### Volume of logs generated

With weekly analysis, the number of jobs logged tends to increase quickly. For a small-sized infrastructure, this volume remains controllable, but it could be an issue for larger infrastructure. In such a case, storing reproducibility logs in a more manageable way could be necessary.

### Technical significance

#### Bioinformatics from the perspective of medical informatics

Bioinformatics analyses have become clinical tools comparable to any other laboratory tests. Consequently, in addition to pre-analytical quality insurance, the NGS analytics pipelines must be standardized and respond to high-level quality requirements. As they are part of the patient care process, the results should be produced by explicitly traceable pipelines and made available without undue delay. Moreover, the overall technical aspects of the pipelines should be transparent for the user. Dockerized tools and pipelines can be archived, shared, restored, and reused easily. In our case, we store the different versions of the tools and of reference datasets (reference genome, etc.) to ensure the reproducibility of results within their original context in the future.

#### New functionalities using the AnalysisManager and the SHINY interface

The AM acts as a connector between the sequencer and the analytics pipeline. Its role is to simplify the management of the data and launch of the analyses. The main interest resides in better and safer data management. The SHINY interface allows the creation of an interactive dashboard, which could provide support for statistical dashboard or visualization, similar to the proposition done by Grüning et al. [43] using Jupyter.

#### Quality, standardization, and reproducibility of the analytical process

Our architecture helps the molecular biologist to control the quality of their results by ensuring a consistent and repeatable analytical process.

- *Traceability*. A metadata file is generated along with the result detailing the input parameters and the version of the tool. We leverage the ReGaTE metadata system [37], which itself uses EDAM [44] ontology for bioinformatics processes.
- *Reproducibility.* All the versions of the tools are stored in our tool registry, allowing us to redeploy a former version if needed. Combined with metadata associated with the results of analysis, this enables the reproducibility of the analysis: we can ensure that we are able to reanalyze data using exactly the pipeline that was used at the time of the initial analysis. Tables 1–3 show traceability and reproducibility logs from the application.
- *Standardization.* AM is combined with the Dockerized tools in Galaxy as an end-to-end pipeline for the user. We strongly reinforce the standardization of the analytical process. We introduced a quality control series along the pipeline, including checksum control after file copy to ensure the integrity of the transfer. Biologists ensure that the results obtained are coherent with other experiments.
- *Microservices*. The architecture presented in this study fits the notion of microservices [45] and container-as-a-service. Microservices help reproducibility by simplifying the exchange of applications, testing processes, and deployment by separating the overall architecture into small independent parts that are easily maintainable.

**Table 1:** Traceability logs of Galaxy tools

Name	Version	workflows	supported	dataHandle	dataDescription	dataFormatEdamOntology
		tools_id	files_id			
samtools_ phase	2.0	samtools_phase_ 2.0.json	3	input_bam	Select dataset to phase	http://edamontology.org/format_ 2572
samtools_ idxstats	2.0	samtools_idxstats_ 2.0.json	4	input	BAM file	http://edamontology.org/format_ 2572

For each tool located on our Galaxy instance, we registered the tool name, the version, and the URI of the Edam ontology, which describe the input and output data of a specific tool.

**Table 2:** Traceability logs of user activities

job_create_time	job_user_email_id	job_tool_id_id	job_params	job_inputs	File_name
2017–05-12T08:51:39.373441	Galaxy user@aphp.fr	samtools_phase_ 2.0.json	{u’chromInfo': u'“/Galaxy-central/tool-data/shared/ucsc/chrom/? .len”“, u’option_set”: u'{“__current_case__”: 1, “min_bq”: “10,” “read_depth”: “242,” “drop_ambiguous”: “False,” “min_het”: “37,” “block_length”: “13,” “option_sets”: “advanced,” “ignore_chimeras”: “False”}“, u’dbkey”: u'“?”'}	{u’input_bam': {u'src': u’hda', u’id': u’f2db41e1fa331b3e', u’uuid': u'5203fb22-cc3c-43f3–9e31–90bd22ded709'}}	MitoChrondrieH. bam
2017–05-12T08:51:20.094488	Galaxy user@aphp.fr	samtools_idxstats_ 2.0.json	{u’chromInfo': u'“/Galaxy-central/tool-data/shared/ucsc/chrom/? .len”“, u’dbkey”: u'“?”'}	{u’input': {u'src': u’hda', u’id': u’f2db41e1fa331b3e', u’uuid': u'5203fb22-cc3c-43f3–9e31–90bd22ded709'}}	MitoChrondrieH. bam
2017–05-12T08:50:44.205220	Galaxy user@aphp.fr	upload1_1.1.4. json	{u’files': u'[{“to_posix_lines”: “Yes,” “NAME”: “None,” “file_data”: “/tmp/nginx_upload_store/ 0000000001,” “space_to_tab”: null, “url_paste”: “", ”__index__“: 0, ”ftp_files“: ”“, ”uuid“: ”None“}]“, u’paramfile”: u'”/export/Galaxy-central/database/files/ tmpkDBRmt““, u’file_type”: u'”auto““, u’files_metadata”: u'{”file_type“: ”auto“, ”__current_case__“: 39}“, u’async_datasets”: u'”None““, u’dbkey”: u'”?"'}	{}	MitoChrondrieH. bam

We store all information related to the execution of a job inside Galaxy. We store the date, the user name, the tool ID, the parameters used, and the name of the inputdata used.

**Table 3:** Traceability of reference genomes

genomeID	version	Description	localization
Hg19plasma	Hg19	Human(Homosapiens):Hg19plasma	/genomes/plasmaMutation/hg19/ hg19.fasta

#### Performance

The use of containers (in our case, the Docker technology) comes at a very low performance cost. According to benchmarks, the use of containers increases the process duration by a minor factor [46]. In our case, because we have limited the interactions between the users and the system, the biologists and technicians of the Molecular Biology platform have to spend reasonable time waiting for results. The AM automatically sends an email once analyses are completed.

#### Scalability

Our proposed architecture is dedicated to small bioinformatics infrastructure. Scalability is not our main goal. However, parallelization and job queuing would be possible using Slurm and/or a Grid Engine Cluster (configurable in Galaxy).

#### Evolutibility

New tools will not interfere with preexisting ones. So as new tools are added or versions updated (e.g., IonTorrentReport V1 vs IonTorrentReport V2) [47], the Dockerized architecture allows an easy simultaneous and prospective comparison between versions for an end-to-end validation of new analysis pipelines.

#### Localization

Many common tools are distributed internationally in English. In a clinical setting, we needed the platform to be accessible in the local language (for us, French). We have translated all the tool-user interfaces in French to avoid any error due to the language barrier.

#### Network of hospitals

The architecture proposed here could help hospitals, and the network of hospitals, to share tools. The main idea is to render any underlying change transparent to the user: the interface remains the same, but the tools can evolve and be updated or shared. A tool, or version of a tool, developed at the Pompidou Hospital could easily be shared as a Docker image with another hospital from the local network of hospital, or more widely.

#### Implementation at HEGP

The molecular biologists use the Galaxy platform to analyze results from NGS, including the identification of variants in the tumor tissue, identification of variants in circulating tumor DNA [48], and copy number analysis [47].

Our platform is deployed on a production server (running on Linux, with 20 cores, and 50 GB of RAM). In our experience, the minimal requirement for development purposes is a machine with 4 cores and 6 GB of RAM. Our AM instance is connected to an Ion-Proton^®^ sequencer.

### Clinical significance

#### Biological significance

We found that our architecture could help respond to several requirements of clinical NGS: the processes are logged and stored. Logs can be reviewed afterward, and containerized applications are archived and can be restarted easily. Our Galaxy platform is used on a daily basis by the biologists and technicians of the Molecular Biology Department. Compared to the situation prior to the deployment of the Analytical Server, users estimated that the implementation of the platform reduced by 60% to 80% the time spent by molecular biologists (manual file transfer, online and offline analysis, reformatting, etc.). For example, before the implementation of the Galaxy AM, the BAM file of each sample was manually downloaded from the sequencer server and renamed. The BAM files were then uploaded to the Galaxy server, and each file was individually selected as an input file for the Plasma mutation analysis pipeline. Once the analysis ended, the resulting files were manually downloaded and renamed for further analysis by the clinical biologist. Batch analysis and automated file renaming, as well as secured file transfer between the sequencer, the analytic server, and the backup server have dramatically improved the time consuming, fastidious, and error-prone task. The AM only reduces the amount of human manipulation and transformation. As such, it does not add any level of complexity. Sample names are deciphered from the sequencer ones to the user-defined sample identifiers; file naming and organization are automated.

#### Feedback from the users

The feedback from molecular biologists is overall very positive. The platform is used on a daily basis by technicians, as well as molecular biologists. The use of both Galaxy and AM reduced the time needed to manipulate the files, and it enables a safer manipulation of the data (we included verification of the integrity of the files after the transfer of BAM files). In Galaxy, the containerization is transparent for the user. The AM enables the backup of the data (directly from the sequencer) and batch treatment of resulting files for non-experts.

From a bioinformatics standpoint, the use of the platform is beneficial as well: the Dockerized tools allowed the easy maintenance, update, and downgrade of the different tools.

From a system administrator standpoint, Dockerization modifies the organization of the workload: in addition to the time needed to develop a new analytic tool, the bioinformaticians need to adapt the tools as a container. The administrator must then produce the Galaxy file describing the tool (an xml file). The containerization step is new and may require time for complex tools. For example, the Dockerization of the analysis of circulated tumors in plasma [49] took 2 days, whereas the Dockerization of Samtools took 10 minutes. Note that many bioinformatics tools have already been Dockerized and shared by the community (e.g., [50, 51]). In which case, only the production of the Galaxy tools description (xml file) is required. In our experience, the time invested during this step is often saved later when deploying the tool in a new setting. Moreover, the containerization prevents any library complex installation or compatibility issue.

Overall, including maintenance, evolution of the platform, and administration, our administrator spent between 2 and 3 hours per week managing the platform, including user training.

However, the microservice architecture requires somewhat more expertise to plan the overall deployment of the different services. The administrators must take into account the communication and the dependencies between the containers.

#### Precision medicine

In medicine, and especially in oncology, the border between clinical care and research is reducing every day. New methods and algorithms are developed in research facilities and need to be quickly deployed in the hospital. The architecture based on Docker can ease the integration of new tools as a single independent container. As such, the improvements in algorithms and pipelines are easily translated into clinical care. Accelerating the availability of NGS results for clinical decisions and making traceability easier and systematic sends a strong signal of evidence for genomic medicine. This increases the quality and speed of clinical decision-making, and consequently the chances for the patient of being prescribed the best treatment without delay. For example, the biologists provided us with an early version of the pipeline for the analysis of circulating tumors’ DNA (“plasmaMutationDetector” [49]). We Dockerized the tool and deployed it on our development platform (a duplicate of our production platform). The tool was used in development for a few months for a research project. When the tool was considered stable after a few cycles of debugging and updates, we deployed the tools “plasmaMutationDetector” on the production server. The deployment was largely simplified because we only needed to copy the container of the new tool. The installation on the production server requires the copy of the files, and rebuilding and restarting the platform.

#### Use case at the European Hospital Georges Pompidou

The molecular biologist uses the Galaxy platform to analyze results from NGS, including the identification of variants in tumor tissue [47], identification of variants in circulating tumor DNA [49], and copy number analysis.

## Conclusion

Workflow management platforms and container technologies can help traceability and reproducibility. In this article, we introduced an architecture based on Galaxy and embedding tools and Docker images. We focused on the management of the data and ensured the possible replication of experiments by tracing tools and arguments. We relied on EDAM ontology for better interoperability. Our architecture is dedicated to small bioinformatics infrastructure and relies on microservices and containers. Our solution is deployed and used at HEGP for routine analysis.

## Availability and requirements

Project name: GalaxyDockerPublic

Project home page: https://github.com/CARPEM/GalaxyDocker

Operating system(s): Ubuntu 14.04

Programming language: Python, shell, R

Other requirements: Docker, Docker-compose

License: MIT

## Abbreviations

AM: analysis manager; API: application programming interface; HEGP: european hospital georges pompidou; NGS: next-generation sequencing

## Availability of supporting data

The Dockerized Galaxy embedding examples of Dockerized tools, ReGaTE traceability metadata creation system, configuration files and code, and the AM module connecting a sequencer to the services of the Galaxy platform directly can be found at the above project homepage. Snapshots are also stored in the *GigaScience* repository, *Giga*DB [52].

## Conflict of interest

The authors declare no conflict of interest.
